# A Comprehensive Expression Analysis of Mucins in Appendiceal Carcinoma in a Multicenter Study: MUC3 Is a Novel Prognostic Factor

**DOI:** 10.1371/journal.pone.0115613

**Published:** 2014-12-31

**Authors:** Hiroaki Shibahara, Michiyo Higashi, Seiya Yokoyama, Karine Rousseau, Iwao Kitazono, Masahiko Osako, Hiroshi Shirahama, Yukie Tashiro, Yasuhiro Kurumiya, Michihiko Narita, Shingo Kuze, Hiroshi Hasagawa, Takehito Kato, Hitoshi Kubota, Hideaki Suzuki, Toshiyuki Arai, Yu Sakai, Norihiro Yuasa, Masahiko Fujino, Shinji Kondo, Yoshichika Okamoto, Tatsuyoshi Yamamoto, Takashi Hiromatsu, Eiji Sasaki, Kazuhisa Shirai, Satoru Kawai, Koutarou Hattori, Hideki Tsuji, Osamu Okochi, Masaki Sakamoto, Akinobu Kondo, Naomi Konishi, Surinder K. Batra, Suguru Yonezawa

**Affiliations:** 1 Department of Palliative Care, Toyota Kosei Hospital, Toyota, Japan; 2 Department of Human Pathology, Field of Oncology, Kagoshima University Graduate School of Medical and Dental Sciences, Kagoshima, Japan; 3 Wellcome Trust Centre for Cell-Matrix Research, Faculty of Life Sciences, University of Manchester, United Kingdom; 4 Department of Surgery, Kagoshima Medical Association Hospital, Kagoshima, Japan; 5 Department of Pathology, Imakiire General Hospital, Kagoshima, Japan; 6 Department of Surgery, Toyota Kosei Hospital, Toyota, Japan; 7 Department of Pathology, Toyota Kosei Hospital, Toyota, Japan; 8 Department of Surgery, Chutoen General Medical Center, Kakegawa, Japan; 9 Department of Surgery, Japanese Red Cross Nagoya Daini Hospital, Nagoya, Japan; 10 Department of Surgery, Toyohashi Municipal Hospital, Toyohashi, Japan; 11 Department of Surgery, Handa City Hospital, Handa, Japan; 12 Department of Surgery, Meijo Hospital, Nagoya, Japan; 13 Department of Surgery, Anjo Kosei Hospital, Anjo, Japan; 14 Department of Pathology, Anjo Kosei Hospital, Anjo, Japan; 15 Department of Surgery, Japanese Red Cross Nagoya Daiichi Hospital, Nagoya, Japan; 16 Department of Pathology, Japanese Red Cross Nagoya Daiichi Hospital, Nagoya, Japan; 17 Department of Surgery, Sakashita Hospital, Nakatsugawa, Japan; 18 Department of Surgery, Shizuoka Saiseikai General Hospital, Shizuoka, Japan; 19 Department of Surgery, Tokai Hospital, Nagoya, Japan; 20 Department of Surgery, Kiryu Kosei General Hospital, Kiryu, Japan; 21 Department of Surgery, Kamiiida Daiichi General Hospital, Nagoya, Japan; 22 Department of Surgery, Yamashita Hospital, Ichinomiya, Japan; 23 Department of Surgery, Tsushima City Hospital, Tsushima, Japan; 24 Department of Surgery, Minami Seikyo Hospital, Nagoya, Japan; 25 Department of Surgery, Toyota Memorial Hospital, Toyota, Japan; 26 Department of Surgery, Tosei General Hospital, Seto, Japan; 27 Department of Surgery, Nagoya Tokushukai General Hospital, Kasugai, Japan; 28 Department of Surgery, Saiseikai Matsusaka General Hospital, Matsusaka, Japan; 29 Department of Surgery, Mie Prefectural General Medical Center, Yokkaichi, Japan; 30 Departments of Biochemistry and Molecular Biology, Buffett Cancer Center, Eppley Institute for Research in Cancer and Allied Diseases, University of Nebraska Medical Center, Omaha, Nebraska, United States of America; King's College London, United Kingdom

## Abstract

**Background:**

Mucins are implicated in survival in various cancers, but there have been no report addressed on survival in appendiceal carcinoma, an uncommon disease with different clinical and pathological features from those of other colon cancers. We aimed to investigate the clinical implications of expression of mucins in appendiceal carcinoma.

**Methods:**

Expression profiles of MUC1, MUC2, MUC3, MUC4, MUC5AC, MUC6, MUC16 and MUC17 in cancer tissue were examined by immunohistochemistry in 108 cases of surgically resected appendiceal carcinoma.

**Results:**

The following relationships of mucins with clinicopathologic factors were identified: MUC1 with positive lymphatic invasion (p = 0.036); MUC2 with histological type (mucinous carcinoma, p<0.001), superficial invasion depth (p = 0.007), negative venous invasion (p = 0.003), and curative resection (p = 0.019); MUC3 with non-curative resection (p = 0.017); MUC5AC with histological type (mucinous carcinoma, p = 0.002), negative lymphatic invasion (p = 0.021), and negative venous invasion (p = 0.022); and MUC16 with positive lymph node metastasis (p = 0.035), positive venous invasion (p<0.05), and non-curative resection (p = 0.035). A poor prognosis was related to positive lymph node metastasis (p = 0.04), positive lymphatic invasion (p = 0.02), positive venous invasion (p<0.001), non-curative resection (p<0.001), and positive expression of MUC3 (p = 0.004). In multivariate analysis, positive venous invasion (HR: 6.93, 95% CI: 1.93–24.96, p = 0.003), non-curative resection (HR: 10.19, 95% CI: 3.05–34.07, p<0.001) and positive MUC3 expression (HR: 3.37, 95% CI: 1.13–10.03, p = 0.03) were identified as significant independent prognostic factors in patients with appendiceal carcinoma.

**Conclusions:**

Expression of MUC3 in appendiceal carcinoma is an independent factor for poor prognosis and a useful predictor of outcome in patients with appendiceal carcinoma after surgery.

## Introduction

Appendiceal cancer is rare in the United States, with an age-adjusted incidence of 0.12 cases per 1,000,000 people per year [1], and a rate among intestinal cancers of 0.7%, compared to 1.5% for small bowel carcinoma and 97.8% for colon carcinoma in the Surveillance, Epidemiology and End Results (SEER) registry [2]. A similar rarity of appendiceal carcinoma is also found in Japan, with incidences of 0.2% in the Japanese Society for Cancer of the Colon and Rectum Registry and 0.08% in the Japanese Autopsy Annual Database of Colorectal Cancer [3]. The disease differs from cancers at other sites in the colon, with clinical presentation of acute abdominal symptoms suggestive of appendicitis [4], [5] and peritoneal mucinous carcinomatosis. The 5-year survival rate for appendiceal carcinoma after surgery is 46–64% [5]–[7]. Curative surgical resection is required for improving survival, and the pathological characteristics of the tumor affect prognosis. Among histological types, patients with non-mucinous carcinoma have poorer survival than those with mucinous carcinoma [5], [8], and those with signet ring cell carcinoma also have poor survival [1]. Cases with a high histological grade have poorer survival than low grade cases [6], [7]. Thus, prognostic factors in appendiceal carcinoma have included curative resection [6], primary tumor status [6], histological type [1], [5], [6], [8], and histological grade [6], [7], [9].

Mucins are high molecular weight glycoproteins having core protein backbones by O-glycosidic linkages with oligosaccharides [10]. Eighteen core proteins for human mucins (MUC1-MUC8, MUC12, MUC13, MUC15-17, MUC19-21) have been identified. The first cloned, MUC1, has been reported to be one of the most important human tumor antigens, namely, the second ranking next to WT1 [11]. Yonezawa et al. showed that MUC1 and/or MUC4 expression is related to a poorer prognosis for various human cancers, whereas MUC2 expression is related to a better prognosis [10], [12]. Aberrant expression of MUC3, MUC4, MUC5AC and MUC6 is found in pancreatic intraepithelial neoplasia [13], [14], and MUC16 and MUC17 are expressed in pancreatobiliary and small intestinal cancers [15]–[17] and have high prognostic value [15], [17]–[19]. Mucin expression also occurs in appendiceal carcinoma [20]–[26], however, there is no study for the relationship between mucin expression and survival of over 100 surgically-treated patients with appendiceal carcinoma.

The aim of this study was to investigate whether expression of mucins (MUC1, MUC2, MUC3, MUC4, MUC5AC, MUC6, MUC16 and MUC17) has prognostic significance in patients with appendiceal carcinoma using surgical specimens collected from multiple centers.

## Materials and Methods

### Patients and Tissue Specimens

Between 1991 and 2013, 108 resected specimens of appendiceal carcinoma were collected from 23 hospitals in Japan: Toyota Kosei Hospital, Kagoshima Medical Association Hospital, Imakiire General Hospital, Chutoen General Medical Center, Japanese Red Cross Nagoya Daini Hospital, Toyohashi Municipal Hospital, Handa City Hospital, Meijo Hospital, Anjo Kosei Hospital, Japanese Red Cross Nagoya Daiichi Hospital, Sakashita Hospital, Shizuoka Saiseikai General Hospital, Tokai Hospital, Kiryu Kosei General Hospital, Kamiiida Daiichi General Hospital, Yamashita Hospital, Tsushima City Hospital, Minami Seikyo Hospital, Toyota Memorial Hospital, Tosei General Hospital, Nagoya Tokushukai General Hospital, Saiseikai Matsusaka General Hospital, and Mie Prefectural General Medical Center.

This study was conducted in accordance with the guiding principles of the Declaration of Helsinki. Informed, written consent was obtained from 10 patients, and was approved by the Ethics Committees of Kagoshima-shi Medical Association Hospital (KMAH 2011-02-02), Japanese Red Cross Nagoya Daini Hospital (IRB20140128-7), Toyota Memorial Hospital (1211-4), and Saiseikai Matsusaka General Hospital (52-2013). For the other patients without informed consent, the Institutional Review Board of Toyota Kosei Hospital (22-ST04), the Ethics Committees of Imakiire General Hospital (119-2013), Toyohashi Municipal Hospital (43-2011), Japanese Red Cross Nagoya Daiichi Hospital (26-2013), Sakashita Hospital (1-2013), Shizuoka Saiseikai General Hospital (25-3-02), Tsushima City Hospital (2013-06), Toyota Memorial Hospital (1211-4), Tosei General Hospital (420-2013), Chutoen General Medical Center, Handa City Hospital, Kiryu Kosei General Hospital, Kamiiida Daiichi General Hospital, and Yamashita Hospital, and the hospital directors of Meijo Hospital, Anjo Kosei Hospital, Tokai Hospital, Minami Seikyo Hospital, Nagoya Tokushukai General Hospital, and Mie Prefectural General Medical Center (no specified number in these eleven hospitals) waived the need for written informed consent from the participants, and gave us their approval for use of the resected specimens, under the strict condition of privacy protection of the personal information of the patients.

Primary appendiceal carcinomas that were clinically and pathologically diagnosed by surgeons and pathologists were included in the study. Possible cecum cancers with invasion of the appendix, metastatic cancer to the appendix, or carcinoid of the appendix were excluded. Samples were collected from 55 males and 53 females with a mean age of 65 years (range 23–95). The surgical procedures are shown as Table 1. Mucinous peritonitis was found in laparotomy in 14 cases. Of the 108 patients, 34 died, and the causes of death were the primary disease in 30, another disease in 3, and an unknown cause in 1. All specimens were fixed in formalin, embedded in paraffin and cut into 4-μm -thick sections for immunohistochemistry (IHC), in addition to hematoxylin and eosin (HE) staining.

**Table 1:** Surgical Procedure.

Procedure	No.patients
Primary resection only	88
Type of colectomy	
Appendectomy	20
Resection of the cecum	3
Ileocecal resection	56
Right colectomy	3
Right hemicolectomy	6
Combined resection	
Rectosigmoid colon	1
Uterus and adnexa	1
Liver	1
Elective resection^a^	20
Type of colectomy	
Ileocecal resection	15
Right colectomy	1
Right hemicolectomy	3
Mucinous tumor resection	1
Combined resection	
Retroperitoneum, uterus, right adnexa and rectum	1
Lymph node dissection	
Performed	81
Not performed	27
Curability	
Curative resection	64
Non-curative resection	41
Unknown	3

a Elective resection after pathological diagnosis of appendiceal carcinoma using the resected specimen at the first surgery.

### Immunohistochemistry

MUC1 was detected by a monoclonal antibody (MAb) DF3 (mouse IgG, Toray-Fuji Bionics, Tokyo, Japan), MUC2 by MAb Ccp58 (Novocastra Reagents, Leica Biosystems, Newcastle Upon Tyne, UK), MUC3 by MAb mMUC3-1 (generated by K. Rousseau and D. M. Swallow), MUC4 by MAb 8G7 (generated by S. K. Batra), MUC5AC by MAb CLH2 (Novocastra), MUC6 by MAb CLH5 (Novocastra), MUC16 by MAb OC125 (Acris Antibodies GmbH, Herford, Germany), and MUC17 by a polyclonal anti-human MUC17 (rabbit IgG, generated by S. K. Batra).

IHC was performed using the immunoperoxidase method. Antigen retrieval was performed using CC1 antigen retrieval buffer (pH8.5, EDTA, 100°C, 30 min, Ventana Medical Systems, Tucson, AZ, USA). Sections were incubated with a primary antibody (DF3 diluted 1∶50, 37°C, 32 min; Ccp58 diluted 1∶200, 37°C, 24 min; 8G7 diluted 1∶3000, 37 °C, 32 min; CLH2 diluted 1∶100, 37°C, 24 min; CLH5 diluted 1∶100, 37°C, 24 min; OC125 diluted 1: 100, 37°C, 24 min; anti-human MUC17 diluted 1: 100) in phosphate-buffered saline (PBS) pH 7.4 with 1% bovine serum albumin, and stained on a Benchmark XT automated slide stainer using a diaminobenzidine detection kit (ultraView DAB, Ventana Medical Systems). For MUC3 staining, sections were treated at 100°C for 10 min in 0.01 M citrate buffer at pH 6.0, and then reduced with 0.01 M dithiothreitol in 0.1 M Tris/HCl buffer (pH 8.0) for 30 min at room temperature and alkylated with 0.025 M iodoacetamide in 0.1 M Tris/HCl buffer (pH 8.0) for 30 min [13], [17], [27]. They were incubated with mMUC3-1 at 4°C for 16 h and stained by avidin-biotin complex method. Reaction products were not present when hybridoma culture medium, normal mouse serum, normal rabbit serum, or PBS was used instead of primary antibodies.

### Evaluation of Staining

The results were evaluated based on the percentage of positively stained carcinoma cells. Staining of the following components was evaluated: membrane and cytoplasm for MUC1 and MUC16; supranuclear area for MUC2; membrane for MUC3, cytoplasm for MUC4, MUC5AC and MUC6; and supranuclear area, cytoplasm and membrane for MUC17. Carcinoma cells are considered to be stained positively when at least one of the components was positive. A tumor was considered positive if more than 5% of carcinoma cells were stained, based on our previous use of 5% as the cutoff for mucin expression [17], [28]–[33].

### Statistical Analysis

Associations between mucin expression profiles and clinicopathological factors were examined by chi-square test. Postoperative survival was calculated using the Kaplan-Meier method. Differences in survival curves were compared by log-rank test. A Cox proportional hazard analysis was used to estimate hazard ratios (HRs) and corresponding 95% confidence intervals (CIs) in multivariate analysis. *P*<0.05 was considered significant.

## Results

### MUC expression in carcinomas

**Figure 1:**
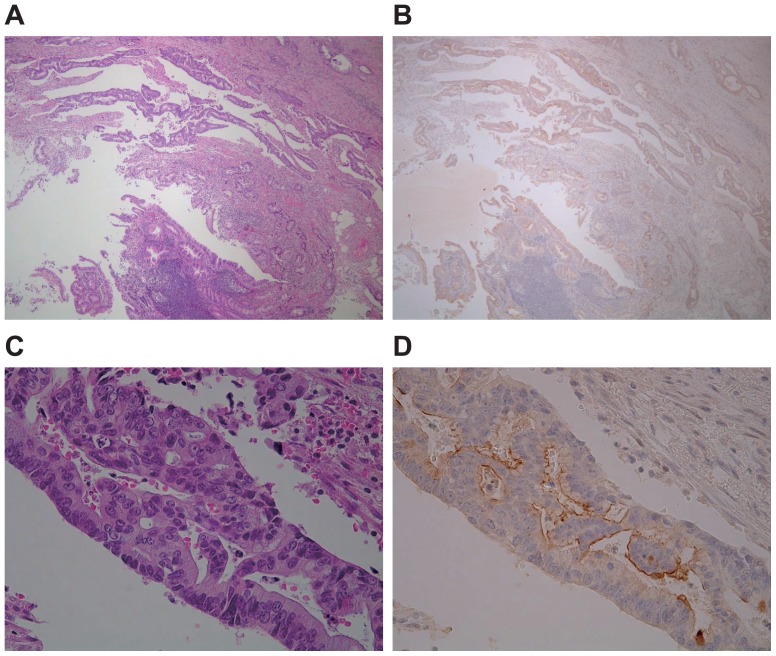
Histological features of appendiceal carcinoma. (A, C) Hematoxylin and eosin stain. (B, D) Immunohistochemistry. MUC3 showed membrane expression in the cell apexes in appendiceal carcinoma.

**Figure 2:**
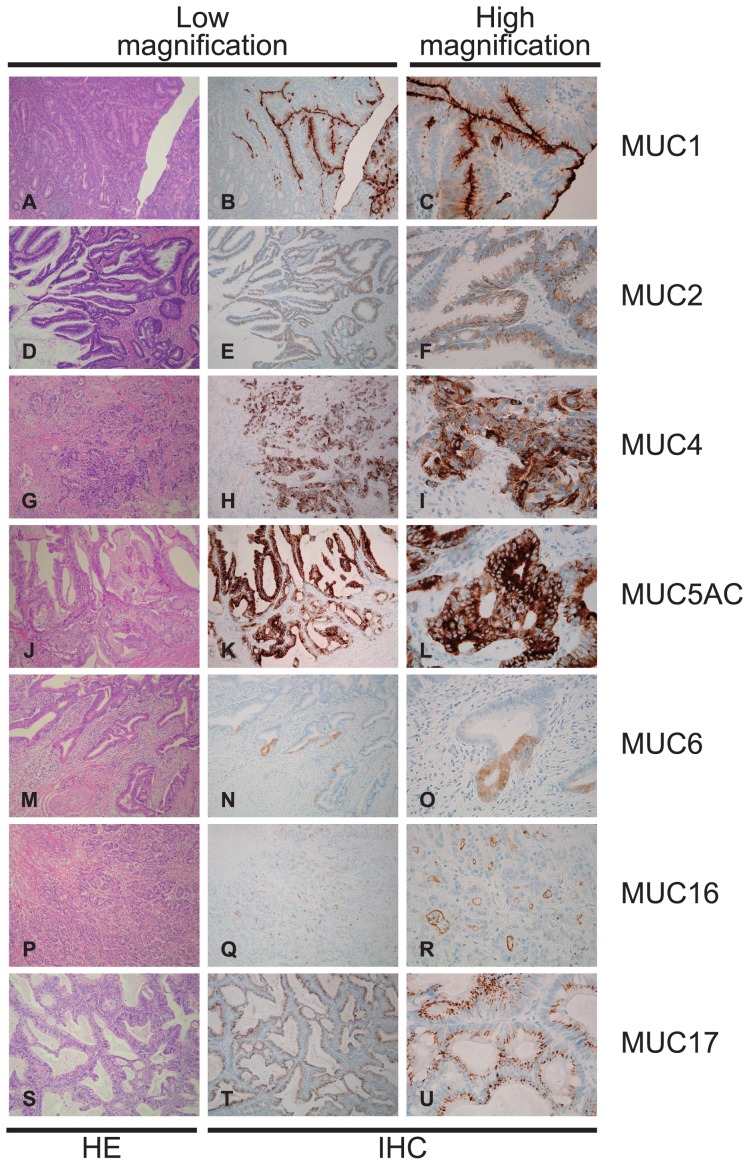
In appendiceal carcinoma cells (A, D, G, J, M, P and S), MUC1 showed membrane expression (B and C); MUC2 showed supranuclear expression (E and F); MUC4 (H and I), MUC5AC (K and L) and MUC6 (N and O) showed cytoplasmic expression; MUC16 showed membrane expression (Q and R); and MUC17 (T and U) showed supranuclear expression. HE, hematoxylin and eosin stain; IHC, immunohistochemical stain.

In the 108 cases, the positive expression rates (more than 5% of carcinoma cells stained) of each mucin antigen were MUC1, 47.2% (51/108); MUC2, 71.3% (77/108); MUC3, 18.5% (20/108); MUC4, 93.5% (101/108); MUC5AC, 50.0% (54/108); MUC6, 4.6% (5/108) MUC16, 16.7% (18/108) and MUC17, 86.1% (93/108). Representative mucin expression patterns in cancer tissues are shown in Fig. 1 (MUC3) and Fig. 2 (MUC1, MUC2, MUC4, MUC5AC, MUC6, MUC16 and MUC17). In appendiceal carcinoma cells, MUC3 showed membrane expression in the cell apexes (Fig. 1A–D); MUC1 showed membrane expression (Fig. 2A–C); MUC2 showed supranuclear expression (Fig. 2D–F); MUC4 (Fig. 2G–I), MUC5AC (Fig. 2J–L), and MUC6 (Fig. 2M–O) showed cytoplasmic expression; MUC16 showed membrane expression (Fig. 2P–R), and MUC17 showed supranuclear expression (Fig. 2S–U).

### Relationship of MUC Expression in Cancer Cells with Clinicopathological Features

Relationships between mucin expression and clinicopathological features are summarized in Table 2. MUC1 expression was related to lymphatic invasion (higher in positive lymphatic invasion, p = 0.036); MUC2 expression was related to histological type (higher for mucinous carcinoma, p<0.001), invasion depth (higher in the superficial area than the musclaris propria, p = 0.007), venous invasion (higher for negative venous invasion, p = 0.003), and curability (higher in curative resection, p = 0.019); MUC3 expression was related to curability (higher in non-curative resection, p = 0.017); MUC5AC expression was related to histological type (higher in mucinous carcinoma, p = 0.002), lymphatic invasion (higher for negative lymphatic invasion, p = 0.021), and venous invasion (higher in negative venous invasion, p = 0.022); and MUC16 expression was related to lymph node metastasis (higher in positive lymph node metastasis, p = 0.035), venous invasion (higher for positive venous invasion, p<0.05), and curability (higher in non-curative resection, p = 0.035).

**Table 2:** Summary of the Data on the Expression of MUC1, MUC2, MUC3, MUC4, MUC5AC, MUC6, MUC16 and MUC17 in Clinicopathological Features of Appendiceal Carcinoma (n = 108).

		MUC1			MUC2			MUC3			MUC4		
Category	No. patients (%)	Negative	Positive	*P* Value	Negative	Positive	*P* Value	Negative	Positive	*P* Value	Negative	Positive	*P* Value
Age (yrs)				0.483			0.132			0.517			0.41
≥65	61 (56.5)	34 (55.7)	27 (44.3)		14 (23)	47 (77)		51 (83.6)	10 (16.4)		5 (8.2)	56 (91.8)	
<65	47 (43.5)	23 (48.9)	24 (51.1)		17 (36.2)	30 (63.8)		37 (78.7)	10 (21.3)		2 (4.3)	45 (95.7)	
Gender				0.126			0.928			0.686			0.659
Men	55 (50.9)	33 (60)	22 (40)		16 (29.1)	39 (70.9)		44 (80)	11 (20)		3 (5.5)	52 (94.5)	
Women	53 (49.1)	24 (45.3)	29 (54.7)		15 (28.3)	38 (71.7)		44 (83)	9 (17)		4 (7.5)	49 (92.5)	
Histological type^a^				0.597			<0.001			0.052			0.111
pap, well, mod	68 (63)	34 (50)	34 (50)		17 (25)	51 (75)		54 (79.4)	14 (20.6)		7 (10.3)	61 (89.7)	
por, sig	19 (17.6)	12 (63.2)	7 (36.8)		14 (73.7)	5 (26.3)		19 (100)	0 (0)		0 (0)	19 (100)	
muc	21 (19.4)	11 (52.4)	10 (47.6)		0 (0)	21 (100)		15 (71.4)	6 (28.6)		0 (0)	21 (100)	
Tumor depth^b^				0.305			0.007			0.103			0.123
m, sm, mp	26 (24.1)	16 (61.5)	10 (38.5)		2 (7.7)	24 (92.3)		24 (92.3)	2 (7.7)		0 (0)	26 (100)	
ss, se, si	82 (75.9)	41 (50)	41 (50)		29 (35.4)	53 (64.6)		64 (78)	18 (22)		7 (8.5)	75 (91.5)	
Lymph node metastasis^c^				0.304			0.056			0.756			0.946
Negative	55 (67.9)	30 (54.5)	25 (45.5)		12 (21.8)	43 (78.2)		45 (81.8)	10 (18.2)		4 (7.3)	51 (92.7)	
Positive	26 (32.1)	11 (42.3)	15 (57.7)		11 (42.3)	15 (57.7)		22 (84.6)	4 (15.4)		2 (7.7)	24 (92.3)	
Lymphatic invasion				0.036			0.083			0.854			0.202
Negative	56 (51.9)	35 (62.5)	21 (37.5)		12 (21.4)	44 (78.6)		46 (82.1)	10 (17.9)		2 (3.6)	54 (96.4)	
Positive	52 (48.1)	22 (42.3)	30 (57.7)		19 (36.5)	33 (63.5)		42 (80.8)	10 (19.2)		5 (9.6)	47 (90.4)	
Venous invasion				0.153			0.003			0.256			0.114
Negative	75 (69.4)	43 (57.3)	32 (42.7)		15 (20)	60 (80)		59 (78.7)	16 (21.3)		3 (4)	72 (96)	
Positive	33 (30.6)	14 (42.4)	19 (57.6)		16 (48.5)	17 (51.5)		29 (87.9)	4 (12.1)		4 (12.1)	29 (87.9)	
Curability^d^				0.164			0.019			0.017			0.831
Curative resection	64 (61)	37 (57.8)	27 (42.2)		13 (20.3)	51 (79.7)		57 (89.1)	7 (10.9)		4 (6.2)	60 (93.8)	
Non-curative resection	41 (39)	18 (43.9)	23 (56.1)		17 (41.5)	24 (58.5)		29 (70.7)	12 (29.3)		3 (7.3)	38 (92.7)	

		MUC5AC			MUC6			MUC16			MUC17		
Category	No. patients (%)	Negative	Positive	*P* Value	Negative	Positive	*P* Value	Negative	Positive	*P* Value	Negative	Positive	*P* Value
Age (yrs)				0.846			0.092			0.931			0.409
≥65	61 (56.5)	30 (49.2)	31 (50.8)		60 (98.4)	1 (1.6)		51 (83.6)	10 (16.4)		7 (11.5)	54 (88.5)	
<65	47 (43.5)	24 (51.1)	23 (48.9)		43 (91.5)	4 (8.5)		39 (83)	8 (17)		8 (17)	39 (83)	
Gender				0.336			0.183			0.263			0.722
Men	55 (50.9)	25 (45.5)	30 (54.5)		51 (92.7)	4 (7.3)		48 (87.3)	7 (12.7)		7 (12.7)	48 (87.3)	
Women	53 (49.1)	29 (54.7)	24 (45.3)		52 (98.1)	1 (1.9)		42 (79.2)	11 (20.8)		8 (15.1)	45 (84.9)	
Histological type^a^				0.002			0.528			0.177			0.105
pap, well, mod	68 (63)	34 (50)	34 (50)		64 (94.1)	4 (5.9)		56 (82.4)	12 (17.6)		11 (16.2)	57 (83.8)	
por, sig	19 (17.6)	15 (78.9)	4 (21.1)		18 (94.7)	1 (5.3)		14 (73.7)	5 (26.3)		4 (21.1)	15 (78.9)	
muc	21 (19.4)	5 (23.8)	16 (76.2)		21 (100)	0 (0)		20 (95.2)	1 (4.8)		0 (0)	21 (100)	
Tumor depth^b^				0.653			0.827			0.841			0.294
m, sm, mp	26 (24.1)	12 (46.2)	14 (53.8)		25 (96.2)	1 (3.8)		22 (84.6)	4 (15.4)		2 (7.7)	24 (92.3)	
ss, se, si	82 (75.9)	42 (51.2)	40 (48.8)		78 (95.1)	4 (4.9)		68 (82.9)	14 (17.1)		13 (15.9)	69 (84.1)	
Lymph node metastasis^c^				0.295			0.325			0.035			0.067
Negative	55 (67.9)	27 (49.1)	28 (50.9)		53 (96.4)	2 (3.6)		50 (90.9)	5 (9.1)		6 (10.9)	49 (89.1)	
Positive	26 (32.1)	16 (61.5)	10 (38.5)		26 (100)	0 (0)		19 (73.1)	7 (26.9)		7 (26.9)	19 (73.1)	
Lymphatic invasion				0.021			0.709			0.228			0.122
Negative	56 (51.9)	22 (39.3)	34 (60.7)		53 (94.6)	3 (5.4)		49 (87.5)	7 (12.5)		5 (8.9)	51 (91.1)	
Positive	52 (48.1)	32 (61.5)	20 (38.5)		50 (96.2)	2 (3.8)		41(78.8)	11 (21.2)		10 (19.2)	42 (80.8)	
Venous invasion				0.022			0.639			<0.05			0.392
Negative	75 (69.4)	32 (42.7)	43 (57.3)		72 (96)	3 (4)		66 (88)	9 (12)		9 (12)	66 (88)	
Positive	33 (30.6)	22 (66.7)	11 (33.3)		31 (93.9)	2 (6.1)		24 (72.7)	9 (27.3)		6 (18.2)	27 (81.8)	
Curability^d^				0.781			0.325			0.035			0.288
Curative resection	64 (61)	31 (48.4)	33 (51.6)		62 (96.9)	2 (3.1)		57 (89.1)	7 (10.9)		11 (17.2)	53 (82.8)	
Non-curative resection	41 (39)	21 (51.2)	20 (48.8)		38 (92.7)	3 (7.3)		30 (73.2)	11 (26.8)		4 (9.8)	37 (90.2)	

a pap, papillary adenocarcinoma; well, well differentiated adenocarcinoma; mod, moderately differentiated adenocarcinoma; por, poorly differentiated adenocarcinoma; sig, signet-ring cell carcinoma; muc, mucinous carcinoma.

b m, mucosa; sm, submucosa; mp, muscularis propria; ss, subserosa, se, serosa; si, invasion to other organ.

c 27 cases without lymph node dissection were excluded.

d 3 cases with unknown details regarding curative or non-curative resection were excluded.

### Relationship of Clinicopathological Factors and Mucin Expression with Survival

**Figure 3:**
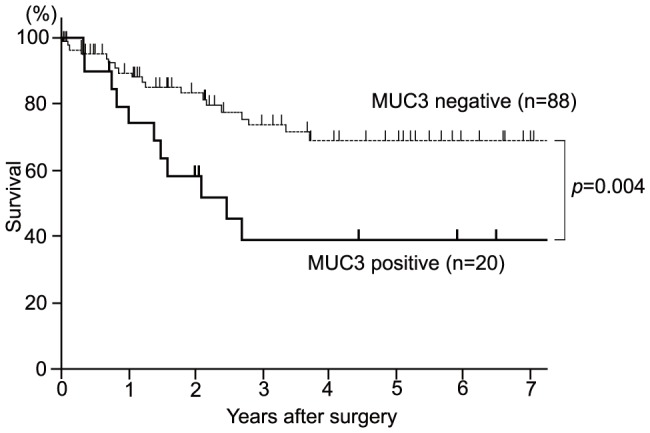
Correlation between mucin expression and the cumulative survival rate. In the study of the correlation between mucin expression and the cumulative survival rate in patients with appendiceal carcinoma using the Kaplan-Meier method, the survival rate of patients with a positive expression of MUC3 were poorer than those of patients with negative expression of MUC3 (p = 0.004).

The 5-year overall survival rate and median survival period were 62.4% and 2.1 years, respectively. Log-rank tests showed that positive lymph node metastasis (p = 0.04), positive lymphatic invasion (p = 0.02), positive venous invasion (p<0.001), and non-curative resection (p<0.001) were significantly related to a worse prognosis (Table 3). Positive expression of MUC3 (p = 0.004) was also significantly related to a worse prognosis (Table 3, Fig. 3), but survival was not correlated with expression of MUC1, MUC2, MUC4, MUC5AC, MUC6, MUC16 and MUC17.

**Table 3:** Survival in Patients with Appendiceal Carcinoma by the Log-Rank Test (n = 108).

	No.patients	5-year survival rate	*P* Value
Category	(%)	(%)	
Age (yrs)			0.054
<65	47 (43.5)	72.4	
≥65	61 (56.5)	53.6	
Gender			0.296
Men	55 (50.9)	57.4	
Women	53 (49.1)	65.9	
Histological type^a^			
pap, well, mod	68 (63)	67.7	0.226
por, sig	19 (17.6)	48.8	
muc	21 (19.4)	60.3	
Tumor depth^b^			0.066
m, sm, mp	26 (24.1)	84	
ss, se, si	82 (75.9)	57.6	
Lymph node metastasis^c^			0.04
Negative	55 (67.9)	76.1	
Positive	26 (32.1)	47.7	
Lymphatic invasion			0.02
Negative	56 (51.9)	77.6	
Positive	52 (48.1)	47	
Venous invasion			<0.001
Negative	75 (69.4)	73.7	
Positive	33 (30.6)	35.4	
Curability^d^			<0.001
Curative resection	64 (61)	83	
Non-curative resection	41 (39)	28.5	
MUC1			0.626
Negative	57 (52.8)	63.1	
Positive	51 (47.2)	62	
MUC2			0.072
Negative	31 (28.7)	51.3	
Positive	77 (71.3)	66.5	
MUC3			0.004
Negative	88 (81.5)	69.1	
Positive	20 (18.5)	38.8	
MUC4			0.467
Negative	7 (6.5)	47.6	
Positive	101 (93.5)	63.4	
MUC5AC			0.433
Negative	54 (50)	59.1	
Positive	54 (50)	66.1	
MUC6			0.698
Negative	103 (95.4)	61.6	
Positive	5 (4.6)	75	
MUC16			0.061
Negative	90 (83.3)	65.4	
Positive	18 (16.7)	48.1	
MUC17			0.5
Negative	15 (13.9)	67.5	
Positive	93 (86.1)	61.9	

a pap, papillary adenocarcinoma; well, well differentiated adenocarcinoma; mod, moderately differentiated adenocarcinoma; por, poorly differentiated adenocarcinoma; sig, signet-ring cell carcinoma; muc, mucinous carcinoma.

b m, mucosa; sm, submucosa; mp, muscularis propria; ss, subserosa, se, serosa; si, invasion to other organ.

c 27 cases without lymph node dissection were excluded.

d 3 cases with unknown details regarding curative or non-curative resection were excluded.

### Multivariate Analysis of Prognostic Factors

The above results identified lymph node metastasis, lymphatic invasion, venous invasion, curative resection and MUC3 expression as candidates for prognostic factors. In multivariate analysis using a Cox proportional hazard model, positive venous invasion (HR: 6.93, 95% CI: 1.93–24.96, p = 0.003), non-curative resection (HR: 10.19, 95% CI: 3.05–34.07, p<0.001), and positive MUC3 expression (HR: 3.37, 95% CI: 1.13–10.03, p = 0.03) were identified as significant independent prognostic factors in patients with appendiceal carcinoma (Table 4).

**Table 4:** Multivariate Analysis of Prognostic Factors.

Category	Hazard Ratio	95% Confidence Interval	*P* Value
Lymph node metastasis			0.511
Negative	1		
Positive	1.41	0.51–3.91	
Lymphatic invasion			0.488
Negative	1		
Positive	1.67	0.39–7.11	
Venous invasion			0.003
Negative	1		
Positive	6.93	1.93–24.96	
Curability			<0.001
Curative resection	1		
Non-curative resection	10.19	3.05–34.07	
MUC3			0.03
Negative	1		
Positive	3.37	1.13–10.03	

## Discussion

In this study, the rates of positive expression were MUC1, 47.2%; MUC2, 71.3%; MUC3, 18.5%; MUC4, 93.5%; MUC5AC, 50.0%; MUC6, 4.6%; and MUC16, 16.7% in 108 cases of appendiceal carcinoma. In colorectal carcinoma, these rates are MUC1, 24–32% [10], [34]; MUC2, 38% [10]; MUC3, 74% [34]; MUC4, 94% [35]; MUC5AC, 34–50% [36], [37]; MUC6, 39% [37]; and MUC16, 64% [18]. The MUC17 expression rate in colon cancer is unknown, but is lower than that in normal epithelium [38]. In appendiceal carcinoma, MUC3, MUC6 and MUC16 had lower expression, MUC2 expression was markedly higher, and MUC1 expression was higher than the respective rates in colorectal carcinoma. These differences indicate the distinct characteristics of appendiceal carcinoma compared to other colorectal cancers. We also previously examined mucin expression in small intestinal carcinoma, and found positive expression rates of MUC1, 51.7%; MUC2, 26.7%; MUC3, 55.0%; MUC4, 51.7%; MUC5AC, 33.3%; MUC6, 10.0%; and MUC16, 8.3% (MUC17 expression was not examined) [17]. Appendiceal carcinoma was MUC1-positive in about half of the cases, similarly to small intestinal carcinoma, but other mucin profiles were different. Thus, with regard to mucin expression, appendiceal carcinoma may have a different carcinogenesis mechanism compared with other colorectal or small intestinal carcinomas.

Expression of MUC3 has been examined in malignancies of the pancreas, periampullary site, bile duct, kidney, salivary gland, lung, and breast, with examining tumor progression and prognosis [13], [39]–[44]. Duncan et al. [34] showed that MUC3 did not affect on survival in colorectal cancer. However, in appendiceal carcinoma, we firstly indicated that MUC3 had impact on survival.

MUC3 maps to a mucin cluster on chromosome 7q22 and is a membrane-bound mucin with tandem repeats of 17 amino acids (HSTPSFTS- SITTTETTS) [10]. IHC of MUC3 (mMUC3-1) in formalin-fixed paraffin-embedded specimens has been developed as a specific method for epitope retrieval [13], [17], [27]. We found that clear linear staining of the surface of villi in the normal mucosa of the small intestine is a good positive control for MUC3 staining [17], [27]. Other studies have used different antibodies, including 1143/B7 [34], [39], [44], [45] and M3P [40], and some have evaluated both membranous and cytoplasmic expression [34], [44], [45]. Using the 1143/B7 antibody, Aloysius et al [39] showed that MUC3 membranous expression is an independent prognostic factor in periampullary cancer. The use of different antibodies and evaluation of different expression patterns might give different results for MUC3, and the association of tumor behavior with results from each MUC3 antibody will be an interesting area for future study.

MUC3 is associated with a poor prognosis in appendiceal carcinoma, but the molecular mechanism of MUC3 in carcinogenesis is uncertain. Epigenetically, expression of MUC3A is contributed by promoter hypomethylation [46]. Cysteine-rich domains of MUC3 promote cell migration and inhibit apoptosis [47], and the MUC3 C-terminal domain undergoes autoproteolysis at its SEA module, which maintains its availability for potentiation of signaling modulated by HER/ErbB2 phosphorylation to promote migration and invasion [48]. Enhanced MUC3 expression by a tetrameric branched peptide with a conserved TFLK motif inhibits bacteria adherence [49], and expression of MUC3 is altered in inflammatory bowel disease and correlated with disease activity and the extent of inflammation [50]. Thus, MUC3 has several potential roles in malignant and inflammatory cells and these effects might be implicated in the poor prognosis of MUC3-positive patients with appendiceal carcinoma.

Expression of MUC1, MUC2, MUC4, MUC5AC, MUC6, MUC16 and MUC17 was not related to survival in appendiceal carcinoma. MUC1 expression is related to a poor prognosis of various human neoplasms and plays an important role in tumor invasion and metastasis [10], [12], but in our cases MUC1 expression was only related to positive lymphatic invasion. Mucinous carcinoma has high MUC2 expression compared to other adenocarcinomas in the pancreas, bile duct, ovary, breast [10], [12] and colorectum [51]. MUC2 expression is also related to a better prognosis of neoplasms in the stomach, pancreas and bile duct [10], [12]. The role of MUC2 in mucinous carcinoma suggests that production of this type of mucin may act as a barrier to cancerous extension, resulting in the indolent nature of many tumors [10]. In the current study, MUC2 expression was associated with mucinous carcinoma, consistent with a previous report [51], and with superficial invasion depth, negative venous invasion, and curative resection, but not with a better prognosis in appendiceal carcinoma.

Shanmugam et al. [35] found that MUC4 expression (≥ 75%) is a poor prognostic factor in colorectal cancer. However, high MUC4 expression (≥ 75%) in appendiceal carcinoma was not significantly related to survival (data not shown). A cut-off value of more than 5% for MUC4 expression detected with antibody 8G7 is significantly related to survival in many tumors [30]–[32], [52]. Kocer et al. [36] found that MUC5AC expression is associated with a better prognosis in colorectal carcinoma, and we also found that MUC5AC expression was related to favorable clinicopathological factors such as negative lymphatic invasion and negative venous invasion. MUC6 expression is a useful marker of pancreatobiliary neoplasms [53]–[55], but has no relationship with clinicopathological factors or survival. MUC16 expression is a poor prognostic factor in cholangiocarcinoma and small intestinal cancer [15], [17], and was related to positive lymph node metastasis, positive venous invasion and non-curative resection in appendiceal carcinoma in the current study.

MUC17 expression is related to tumor progression in pancreatic cancer [19], but was not related to clinicopathological factors or survival in appendiceal carcinoma. MUC17 and MUC3 are similarly expressed on the apical surface of intestinal epithelia, are both present in glycocalyx, and are both located on chromosome 7q22 [56], [57]. *MUC17* and *MUC3A* both have promoter methylation sites, but those are different (−179 to +52 in *MUC17* and −345 to −75 in *MUC3A*) [58]. Regarding histone modification, histone H3-K9 is more highly acetylated in MUC17-positive cells, whereas H3-K9 does not play a critical role in *MUC3A* regulation [58]. In the molecular structures, MUC17 and MUC3 both have an N-terminal large mucin domain, a SEA domain, a transmembrane domain, a cytoplasmic tail, and PDZ-binding motifs [59], [60], but their molecular function is different. In enterocytes, in response to carbachol, MUC17 is relocated from the apical membrane to an intracellular vesicular pool distinct from classical endosomes; this behavior is specific for MUC17, and does not occur for MUC3 [60], [61]. The current study showed a different IHC staining pattern, with MUC17 in the supranuclear area and MUC3 in the membrane, and different clinical significance. The differences in biological behavior between MUC17 and MUC3 may be due to differences in promoters and regulators, or in the structure and domains. Further studies are needed to determine the differences in the roles of these mucins in carcinogenesis.

We emphasize that this study is base on a large collection (n = 108) of a very rare appendiceal carcinoma. Furthermore, we prove that MUC3 only affected the survival, while other mucins with prognostic potentials in many malignancies have little importance. These new data would have a significant clinical impact. The patients with positive MUC3 expression of appendiceal carcinoma, should be followed-up carefully after surgery.

In conclusion, we found that expression of MUC3 in appendiceal carcinoma is an independent poor prognostic factor. MUC3 is a useful predictor of outcome in patients after surgery, and the key mucin for tumor progression in this rare tumor.
